# Dynamic synthetic-scanning photoacoustic tracking monitors hepatic and renal clearance pathway of exogeneous probes in vivo

**DOI:** 10.1038/s41377-024-01644-6

**Published:** 2024-10-31

**Authors:** Jing Lv, Hengrong Lan, Aoji Qin, Tong Sun, Dan Shao, Fei Gao, Junjie Yao, Kamran Avanaki, Liming Nie

**Affiliations:** 1 Guangdong Cardiovascular Institute, Guangdong Provincial People’s Hospital, Guangdong Academy of Medical Sciences, Guangzhou, 510080 China; 2 Medical Research Institute, Guangdong Provincial People’s Hospital, Guangdong Academy of Medical Sciences, Southern Medical University, Guangzhou, 510080 China; 3 School of Biomedical Engineering, Tsinghua University, Beijing, 100084 China; 4 Department of PET Center, Guangdong Provincial People’s Hospital, Guangdong Academy of Medical Sciences, Southern Medical University, Guangzhou, 510080 China; 5 Hybrid Imaging System Laboratory, School of Information Science and Technology, ShanghaiTech University, Shanghai, 201210 China; 6 Department of Biomedical Engineering, Duke University, Durham, NC 27708 USA; 7 Richard and Loan Hill Department of Biomedical Engineering, University of Illinois at Chicago, Chicago, IL 60607 USA

**Keywords:** Photoacoustics, Imaging and sensing

## Abstract

Advancements in precision medicine necessitate understanding drug clearance pathways, especially in organs like the liver and kidneys. Traditional techniques such as PET/CT pose radiation hazards, whereas optical imaging poses challenges in maintaining both depth penetration and high resolution. Moreover, very few longitudinal studies have been performed for drug candidates for different symptoms. Leveraging non-ionizing photoacoustic tomography for deep tissue imaging, we developed a spatiotemporally resolved clearance pathway tracking (SRCPT) method, providing unprecedented insights into drug clearance dynamics within vital organs. SRCPT addresses challenges like laser fluence attenuation, enabling dynamic visualization of drug clearance pathways and essential parameter extraction. We employed a novel frequency component selection based synthetic aperture focusing technique (FCS-SAFT) with respiratory-artifacts-free weighting factors to enhance three-dimensional imaging resolutions. Inspired by this, we investigated the clearance pathway of a clinical drug, mitoxantrone, revealing reduced liver clearance when hepatic function is impaired. Furthermore, immunoglobulin G clearance analysis revealed significant differences among mice with varying renal injury degrees. The accuracy of our method was validated using a double-labeled probe [^68^Ga]DFO-IRDye800CW, showing a strong positive correlation between SRCPT and PET. We believe that this powerful SRCPT promises precise mapping of drug clearance pathways and enhances diagnosis and treatment of liver and kidney-related diseases.

## Abstract (web summary)

The SRCPT method enables dynamic visualization of drug clearance pathways with high precision, particularly in cases of liver and kidney dysfunction, enhancing diagnosis and treatment outcomes.

## Graphical abstract

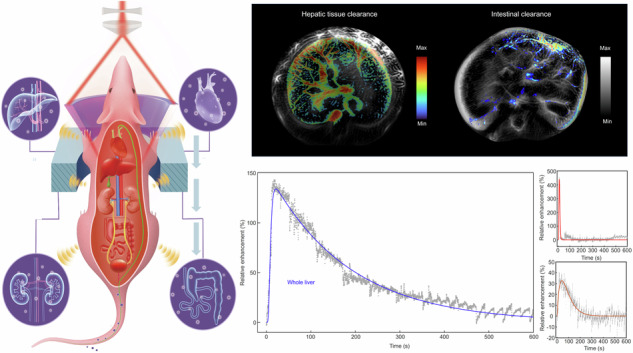

## Introduction

Advancements in precision medicine require a thorough understanding of the clearance pathways of drugs, particularly within vital organs such as the liver and kidneys^1^. This understanding is critical for tailoring interventions and treatments with precision. The significance of achieving high spatiotemporal-resolution monitoring in the clearance pathways of drug cannot be overstated in this case. In recent years, small-animal imaging has become as an indispensable technique for noninvasively studying clearance pathways of drug. These imaging modalities include positron emission tomography/computed tomography (PET/CT)^2^, single photon emission computed tomography/computed tomography (SPECT/CT)^3^, and optical imaging^4,5^, permitting in vivo clearance studies of agents by labeling them with radionuclides or optical tracers. Unfortunately, PET/CT or SPECT/CT employs ionizing radiation, which may bring potential radiation hazards for subjects and hinder longitudinal monitoring.

In vivo fluorescent imaging constitutes the principal optical imaging tool but is limited by its shallow penetration depth and low spatial resolution^6–8^. Thus, the challenge remains to develop an ideal imaging method to study the in vivo clearance of the drug at high spatiotemporal resolution across all organs throughout the entire body.

Photoacoustic tomography (PAT) is a biomedical imaging modality from laboratory to the clinics^9–12^. This technique employs laser excitation and acoustic detection to achieve both superior spatial resolution and deep imaging depth within biological tissues^13–15^.

Our approach, spatiotemporally SRCPT method, leverages deep-penetrating PAT to provide unprecedented insights into the dynamics of probe clearance within the liver and kidneys. The SRCPT incorporated with a Monte Carlo-corrected empiric mathematic model (MC-EMM) algorithm to addresses inherent challenges, such as laser fluence attenuation, ensuring optimal imaging depth and resolution. Through MC-EMM algorithm, SRCPT facilitates dynamic visualization of the clearance properties of probes within specific organs. As a result, we were able to extract valuable parameters such as the peak time (T_max_), the half-life (T_1/2_), the average uptake rate (U_α_), and the average excretion rate (E_β_), and area under the curve (AUC). Analyzing and comparing these parameters could not only aid in identifying the primary metabolic target organs of the agents but also reflect the pathological and metabolic characteristics of tissues or organs.

We investigated the clearance pathway of the clinical drug mitoxantrone in mice, systematically quantifying its drug clearance rates in the liver and kidneys and compared the changes in clearance parameters before and after hepatic and renal function impairment. The results indicated that mitoxantrone is primarily metabolized in the liver, with only a small portion undergoing renal metabolism, when hepatic function is impaired, the hepatic metabolism rate significantly decreases. In the visualization of three-dimensional (3D) data, limitations imposed by the low numerical aperture (NA) of our ultrasound transducer resulted in poor elevation resolution and contrast in 3D data obtained through traditional slice stacking methods. To address this issue, we proposed an frequency component selection based synthetic aperture focusing technique (FCS-SAFT) algorithm based on SAFT^16^, utilizing spectrum separation technology to separate signals of different frequencies. Compared to conventional methods, the FCS-SAFT algorithm significantly improves the all-directions resolution of 3D data and effectively separates high-frequency and low-frequency components representing vascular and tissue signals, respectively.

By optically labeling biomacromolecule immunoglobulin G (IgG), we obtained the clearance characteristics of IgG in the kidneys. Our results revealed statistically significant differences in the clearance of IgG among mice with varying degrees of renal injury. Finally, To validate the accuracy of this method, we synthesized a double-labeled probe [^68^Ga]DFO-IRDye800CW. The compound was dynamically examined for SRCPT and PET in mice. The results showed that there was a strong positive correlation between the two methods.

High spatiotemporal resolution is crucial for precisely mapping the clearance pathways of drug. Through comprehensive investigations, we demonstrate the efficacy of SRCPT in providing detailed information on the pathways and efficiency of drug elimination within the liver and kidneys. In conclusion, SRCPT not only offers real-time insights into the dynamics of drug clearance within vital organs but also opens new frontiers in precision medicine.

## Results

### High spatiotemporal-resolution clearance pathway tracing of small molecules in the liver and kidneys

**Fig. 1:**
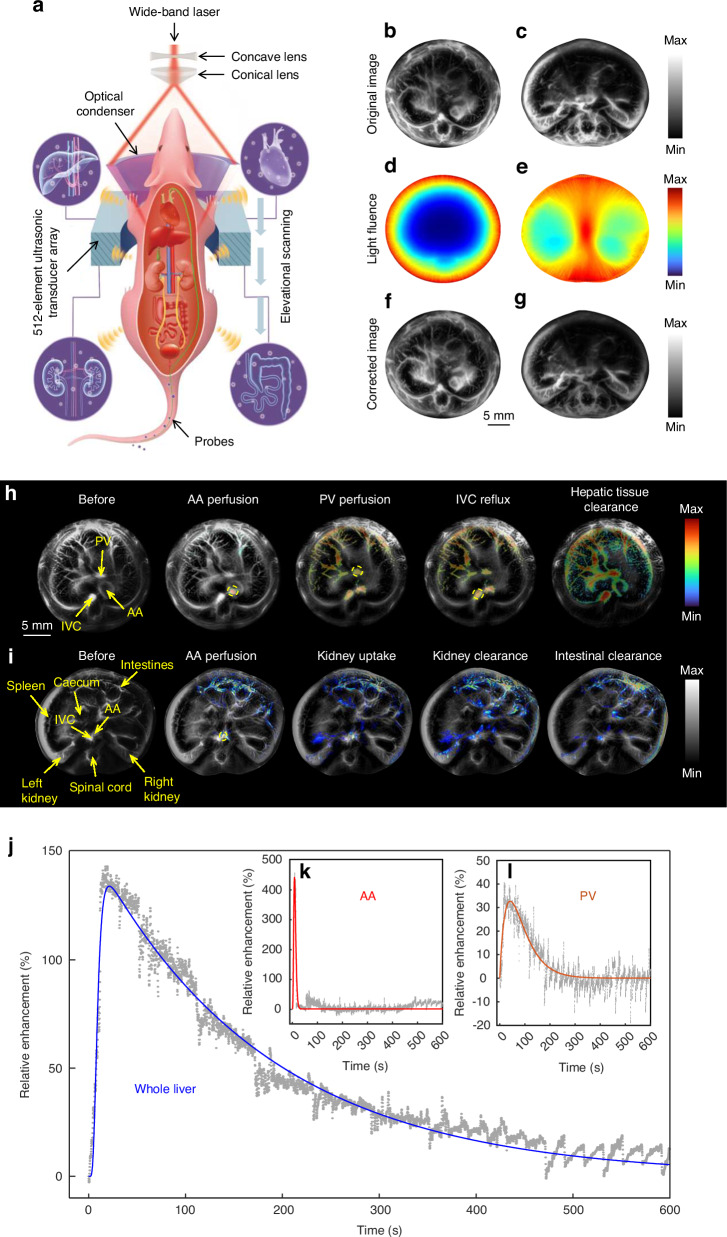
SRCPT with MC-EMM of the small-molecule A1094 in mouse liver at 1064 nm. **a** Schematic diagram of SRCPT of probes in mouse. **b**, **c** The original images of liver and kidney sections. **d**, **e** Light fluence correction of liver and kidney sections using Monte Carlo simulation. **f**, **g** The corrected images of liver and kidney sections. **h** SRCPT of A1094 perfusion in the liver at 0, 7, 15, 20, 30, and 40 s, respectively. Yellow arrows indicate the abdominal aorta (AA), portal vein (PV), and inferior vena cava (IVC). The yellow dashed circles represent the perfusion of A1094 in the AA, PV and IVC, respectively. **i** SRCPT images of A1094 perfusion in the kidneys and intestines at 0, 6, 10, 20, 25, and 30 s, respectively. Yellow arrows indicated the AA, IVC, left kidney, right kidney, intestines, spleen, caecum and spinal cord. **j**–**l** The raw and fitted relative enhancements versus time computed from the quantitative PA signals of A1094 in the AA, PV, and whole liver (*n* = 5), respectively

The hardware schematic for SRCPT is depicted in Fig. 1a. Considering the issue of light attenuation in deep tissue, we compensated for the light flux to analyze the distribution of probes in tissues more accurately. We manually segmented the main organs from the original photoacoustic (PA) images (Fig. 1b, c). We then applied Monte Carlo simulations to calculate the light fluence within these tissues by assigning optical absorption and scattering parameters that represent tissue property variations (Fig. 1d, e)^17,18^, resulting in enhanced contrast in deeper tissue layers and more precise quantification in the corrected images (Fig. 1f, g and Supplementary Videos 1 & 2). Based on the estimated photon flux distribution, we eliminated differences in photon flux by compensating the PA data. Calculations of the signal-to-background ratio (SBR) before and after compensation revealed a ~ 50% increase in SBR for deep tissue in regions with dense vascular distribution.

The vascular distribution of a living mouse was clearly observed at 1064 nm, including the abdominal aorta (AA), portal vein (PV), inferior vena cava (IVC), left kidney, right kidney, intestines, spleen, caecum, spinal cord, and even the intricate vascular network within the liver and kidneys (marked by yellow arrows in Fig. 1h, i). The liver and kidneys play crucial roles as pivotal metabolic organs to exogeneous probes. It is a notable demand for comprehensive clearance investigations to track the intricate behavior of those probes within these anatomical structures^19,20^. We then visualized the clearance of small molecule A1094 in liver and kidneys of mouse by SRCPT. SRCPT accurately monitored the perfusion of A1094 from AA and PV to the liver (Fig. 1h and Supplementary Video 3) and the perfusion of A1094 from AA to the whole kidneys (Fig. 1i and Supplementary Video 4).

To quantify the clearance curve, the outliers caused by breathing were filtered. We employed the MC-EMM algorithm to describe the variation of contrast agent concentration over time. The specific calculation steps include (1), (2), and (3):

1 $${\rm{RE}}({\rm{t}})=[({\rm{PA}}({\rm{t}})-{\rm{PA}}(0))/{\rm{PA}}(0)]\times 100( \% )$$

where PA(t) is the PA intensities of the tissues after injection of probes and PA(0) is the average pre-injection PA intensities. The time course of RE was then fitted using an empiric mathematic model^21^.

2 $${\rm{RE}}({\rm{t}})=\left\{\begin{array}{cc}0&{0\le t < {t}_{0}}\\ A\cdot \left[1-{e}^{-\alpha (t-{t}_{0})}\right]^{q}\cdot {e}^{-\beta (t-{t}_{0})}&{{t}_{0}\le t}\end{array}\right.$$

Considering the light fluence in different time points, we could correct the effect of light fluence by Monte Carlo simulations in empirical mathematical model. ${F}_{{ratio}}$ was the ratio of post injection light fluence to pre-injection light fluence at a certain location.

3 $${\rm{RE}}({\rm{t}})=\left\{\begin{array}{cc}0&{0\le t < {t}_{0}}\\ \frac{A}{{F}_{{ratio}}}\cdot \left[1-{e}^{-\alpha (t-{t}_{0})}\right]^{q}\cdot {e}^{-\beta (t-{t}_{0})}&{{t}_{0}\le t}\end{array}\right.$$

The modified clearance algorithm described above takes light attenuation into account in PAT, making it suitable for the assessment of clearance parameters using SRCPT. Moreover, data acquisition of PAT is much faster than magnetic resonance imaging (MRI), leading to a more accurate quantification. *A/*${F}_{{Ratio}}$ is the upper amplitude of RE, defined as RE_max_. t_0_ is the rise time point. U_α_ is the average uptake rate. E_β_ is the average excretion rate. q affects the slope of early uptake. Finally, the time to maximum RE(T_max_) = max (RE) and the half-life of the elimination RE(T_max_ +T_1/2_) = 0.5×max (RE) were calculated from the fitted curve.

The time courses of the whole liver, AA and PV was fitted using the MC-EMM algorithm. Figure 1j showed that the MC-EMM provided excellent fits to data acquired from PAT as an ideal tool to analyze the clearance of probes. We acquired the following clearance parameters in different functional regions. T_max_ was measured to be 37.61 ± 9.52 s, T_1/2_ was 128.53 ± 15.32 s; U_α_ was 15.62 ± 3.60 min^−1^, E_β_ was 0.039 ± 0.012 min^−1^, AUC_0-600 s_ was 25136 ± 986 of A1094 in the whole liver (Fig. 1j). T_max_ was 5.41 ± 1.02 s in AA (Fig. 1k) and 41.27 ± 10.24 s in PV (Fig. 1l), respectively. From these parameters, we inferred that the A1094 exhibited rapid perfusion from the AA to the liver, with only a minimal portion entering the liver through the PV at a much slower rate, thus emphasizing the dual blood supply characteristic of the liver^22^.

Similarly, we calculated the clearance parameters of A1094 in the kidneys. The results showed that T_max_ was 8.66 ± 2.14 s and T_1/2_ was 10.02 ± 1.04 s; U_α_ was 0.025 ± 0.019 min^−1^ and E_β_ was 0.019 ± 0.021 min^−1^ of A1094 in the kidneys. By analyzing the clearance parameters of A1094 in the liver and kidneys, we found that metabolic T_1/2_ of A1094 in liver was ~13-fold than that in kidneys. The U_α_ of A1094 in liver was ~630-fold than that in kidneys. These findings substantiated that A1094 was primarily metabolized within the liver rather than the kidneys. The quantitative results obtained from the intestines affirm that A1094 was distributed in liver and then excreted into the intestinal tract via the biliary route. By showcasing the dynamic behaviors of probes, this technique serves to highlight their principal sites of metabolism within various organs.

### Clearance pathway of the mitoxantrone at a 3D level

**Fig. 2:**
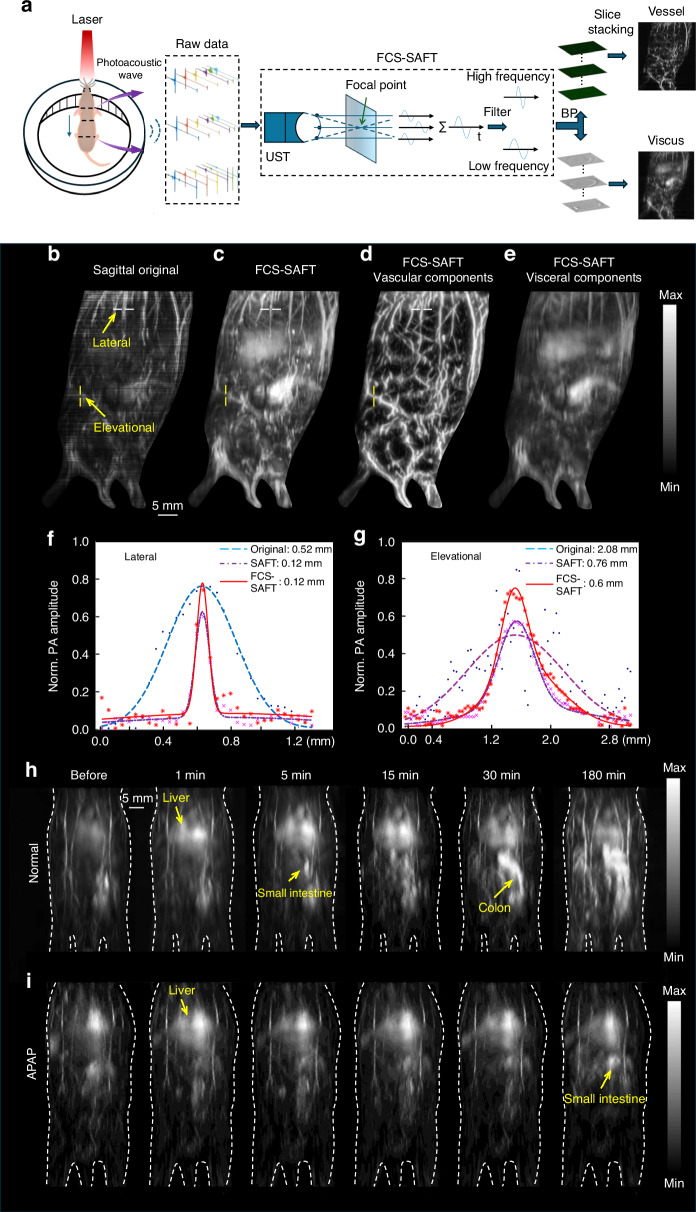
High resolution 3D PA imaging of mitoxantrone in mice. **a** Processing flow of 3D PA data. **b** PA maximum amplitude projection (MAP) image of the 3D image in the sagittal plane of mice without FCS-SAFT algorithm processing in mice. **c** PA MAP image of the 3D image in the sagittal plane of mice with FCS-SAFT algorithm processing in mice. **d** 3D-MAP image of high-frequency vascular component signals obtained after frequency domain separation. **e** 3D-MAP image of low-frequency tissue organ component signals obtained after frequency domain separation. **f** Quantification of lateral resolution by half maximum width (FWHM) from 3D original data, data processed by SAFT and FCS-SAFT algorithm. **g** Quantification of elevation resolution with FWHM from 3D original data, data processed by SAFT and FCS-SAFT algorithm. **h** 3D clearance pathway tracking of mitoxantrone in normal mice. **i** 3D clearance pathway tracking of mitoxantrone in mice with APAP-ALI (*n* = 5 in each group)

Mitoxantrone is an FDA-approved drug for the treatment of malignant lymphoma, breast cancer, and various types of acute leukemia^23^. In this study, we first investigated the clearance characteristics of mitoxantrone in normal liver and liver with acute injury of mice through SRCPT at a 3D level. We utilized an innovative FCS-SAFT incorporating respiratory-artifact-free weighting factors to improve 3D imaging resolution (Fig. 2a–e). The metabolic process is illustrated by the maximum value projection images of 3D PA data in the y-z direction. Measurements of the full-width at half-maximum (FWHM) of the image data indicate that, compared to the original data, the data processed with the FCS-SAFT algorithm show a 4-5 times improvement in lateral resolution and a 2–3 times enhancement in elevation resolution (Fig. 2f, g). Moreover, compared to the SAFT algorithm, this approach yields higher contrast in vascular components and clearer representation of tissue organ components.

The low-frequency PA signals from mouse tissue organs indicated that within the same timeframe, in normal mice, mitoxantrone was primarily metabolized in the liver, followed by rapid excretion from the liver into the small intestine, and subsequently reaching the large intestine. However, in the group with acute liver injury induced by acetaminophen (APAP-ALI), due to impaired liver function, we observed that mitoxantrone was not metabolized quickly, with only a small portion being excreted into the small intestine (Fig. 2h–i and Supplementary Video 5). The above results demonstrated that by combining the SRPCT method with our developed FCS-SAFT algorithm, we are able to clearly trace the dynamic clearance process of drugs in mice. This could serve as a tool for tracking other drugs in the future.

### Spatiotemporally resolved clearance pathway tracking of a clinical drug mitoxantrone

**Fig. 3:**
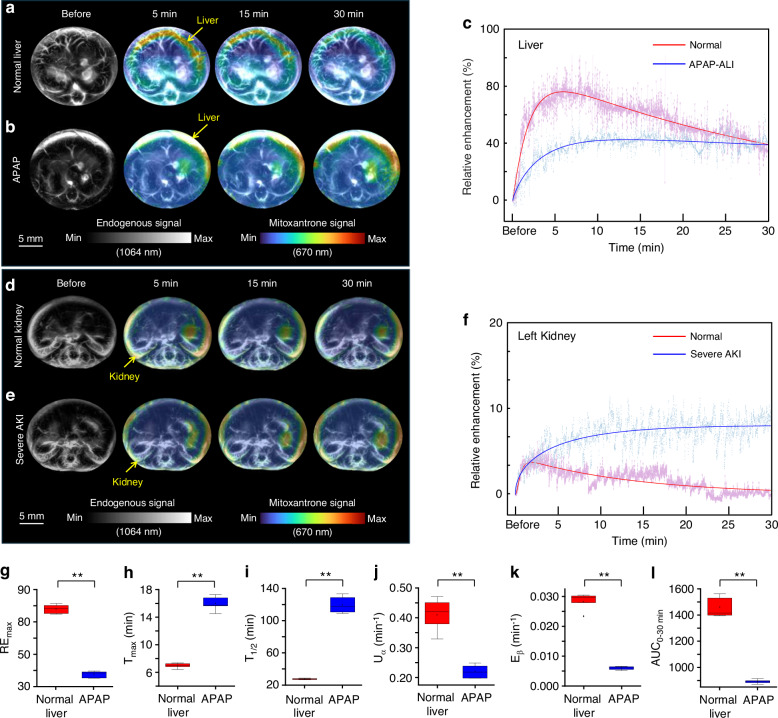
SRCPT of mitoxantrone. **a** PA signal distribution of mitoxantrone in normal mouse liver at different time points. **b** PA signal distribution of mitoxantrone in APAP-ALI mouse liver at different time points. **c** The raw and fitted relative enhancements versus time computed from the quantitative PA signals of mitoxantrone in normal and APAP-ALI liver, respectively. **d** PA signal distribution of mitoxantrone in normal mouse kidneys at different time points. **e** PA signal distribution of mitoxantrone in the mouse kidneys with AKI at different time points. **f** The raw and fitted relative enhancements versus time computed from the quantitative PA signals of mitoxantrone in normal and severe AKI kidney, respectively. **g**–**l** Quantitative comparison of multiple clearance parameters (RE_max_, T_max_, T_1/2_, U_α_min^−1^, E_β_, and AUC_0-30 min_) of mitoxantrone in the normal liver group and the APAP mouse group (*n* = 5 in each group). *p < 0.05, **p < 0.01

On the basis of 3D imaging, we further explored the clearance characteristics of mitoxantrone in the main metabolic organs, the liver and kidneys. We observed that in normal liver, mitoxantrone reached its peak drug concentration approximately 5 min after intravenous injection, followed by rapid metabolism within 30 min. In the group with APAP-ALI, mitoxantrone did not undergo rapid metabolism after entering the mouse liver but accumulated gradually with slight excretion after 15 min (Fig. 3a-c). By using the MC-EMM algorithm, we obtained the clearance parameters of mitoxantrone in normal mouse liver. RE_max_ was measured to be 76.89 ± 2.57%, T_max_ was 6.77 ± 0.54 min, T_1/2_ was 28.87 ± 0.69 min, U_α_ was 0.43 ± 0.06 min^−1^, E_β_ was 0.03 ± 0.0004 min^−1^, and AUC_0-30 min_ was 1418.30 ± 130.31. In parallel, we calculated the clearance parameters of APAP-ALI: RE_max_ was measured to be 38.37 ± 1.88%, T_max_ was 15.48 ± 1.48 min, T_1/2_ was 117.83 ± 18.74 min; U_α_ was 0.22 ± 0.02 min^−1^, E_β_ was 0.0061 ± 0.00085 min^−1^, and AUC_0-30 min_ was 888.56 ± 15.66 (Fig. 3g-l).

By analyzing the above parameters, mitoxantrone in APAP-ALI group reached its peak concentration about 5 min later than in normal liver, and the maximum concentration is only 50.0% of that in normal liver. The T_1/2_ of the drug in the liver is approximately 4 times longer than in normal mice. The U_α_ and E_β_ of mitoxantrone in normal liver are ~2 times and ~5 times greater than those with APAP-ALI, respectively. The results indicated that during APAP-ALI the liver capacity for drug absorption and elimination is severely impaired, leading to a significant disruption of drug metabolism in the liver.

We also investigated the clearance characteristics of mitoxantrone in normal kidneys and kidneys with acute renal injury through SRCPT. We observed that in normal kidney, mitoxantrone reached its peak drug concentration approximately 2 min after intravenous injection, followed by rapid metabolism within 30 min. In the group with severe acute kidney injury (AKI), mitoxantrone gradually accumulated in the mouse kidneys without excretion within 30 min (Fig. 3d, e). By using the MC-EMM algorithm, we obtained the clearance parameters of mitoxantrone in normal kidney: RE_max_ was measured to be 4.50 ± 0.84%, T_max_ was 2.02 ± 0.13 min, T_1/2_ was 10.81 ± 0.34 min; U_α_ was 2.04 ± 0.31 min^−1^, E_β_ was 0.07 ± 0.002 ms^−1^, AUC_0-30_ _min_ was 53.58 ± 13.26. In severe AKI, we only calculated U_α_ of mitoxantrone at 0.12 ± 0.01 min^−1^ because there was no declined trend of the curve within 30 min (Fig. 3f).

We found that RE_max_ of mitoxantrone in the liver was ~35 times higher in normal liver than that in the normal kidneys and that AUC_0-30_ _min_ of mitoxantrone in the liver was ~53 times higher in normal liver than that in the normal kidneys. These indicated that most mitoxantrone was metabolized through the liver but only a small portion through the kidneys. The U_α_ of mitoxantrone in normal kidney are ~17 times greater than that with severe AKI. Due to severe kidney injury, mitoxantrone could not be excreted from the kidneys within 30 min, leading to the continuous accumulation of mitoxantrone. The above results indicate that the absorption capacity of kidney in severe renal injury still retains some functionality while the excretion capability is severely damaged.

### Clearance tracing of tracer labelled biomacromolecules in the injured kidney

Many diseases are caused by the abnormal deposition of biomacromolecules such as specific proteins^24,25^. For example, the deposition of IgG in the kidneys is closely associated with glomerulonephritis^26^, a common kidney disorder. In view of this, we applied SRCPT with MC-EMM algorithm to evaluate the clearance characteristics of IgG in kidneys. The results showed that we could visualize the dynamic deposition of human IgG in kidneys of mice with no, mild, and severe AKI (Supplementary Video 6 & 7). We used BALB/c nude mice as study subjects because they are immunodeficient and less prone to elicit humoral immune responses, making them suitable for observing IgG deposits including human IgG in different species. Human IgG was first labelled with ICG by amidation reaction (see Methods). The molecular weight of ICG is 0.83 kD, accounting for only 0.55% of the molecular weight of IgG (150 kD), therefore having a negligible effect on the clearance properties of IgG.

**Fig. 4:**
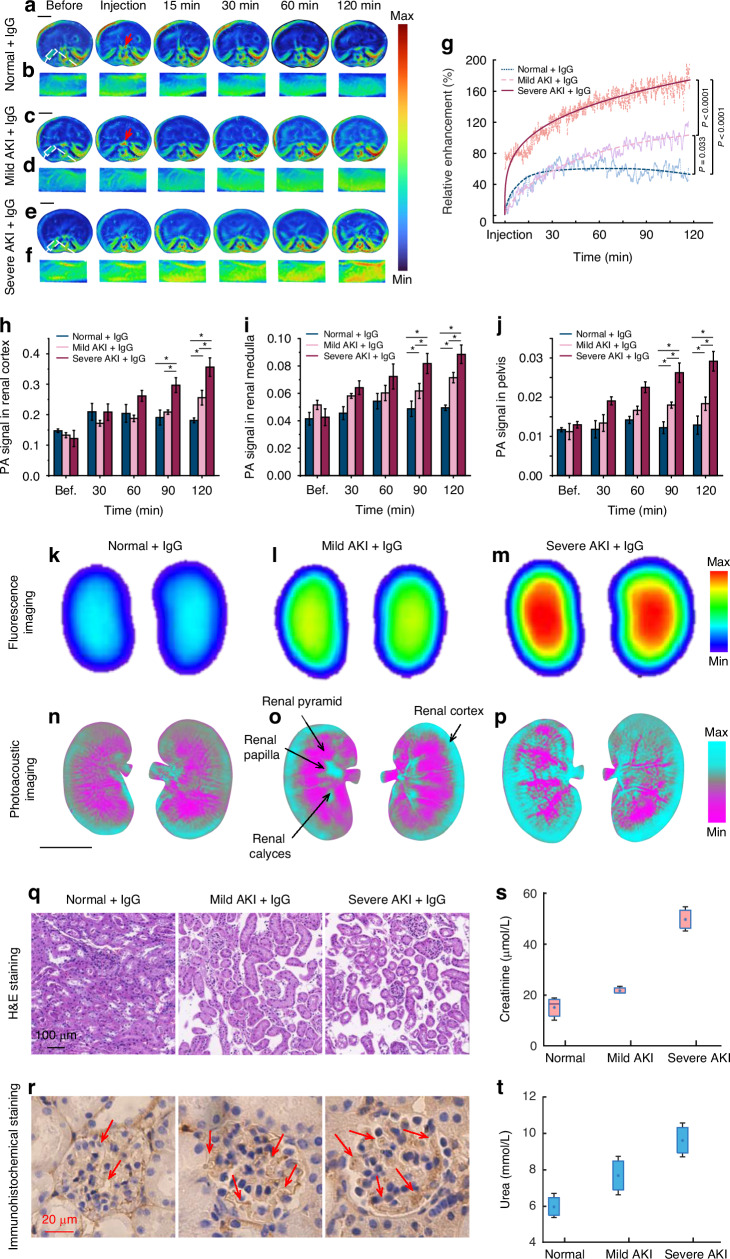
SRCPT monitors the deposition of ICG-labelled IgG (ICG-IgG) in the kidneys of normal mice and mice with mild AKI or severe AKI at 780 nm. **a** Real-time SRCPT snapshots of ICG-IgG deposition in the kidneys of normal mice at different time points in vivo (*n* = 5). **b** Local magnification in the left kidney of the mouse iq22n1q part of **a**. **c** Real-time SRCPT snapshots of ICG-IgG deposition in the kidneys of mice with mild AKI at different time points in vivo (*n* = 5). **d** Local magnification in the left kidney of the mouse in part of (**c**). **e** Real-time SRCPT snapshots of ICG-IgG deposition in the kidneys of mice with severe AKI at different time points in vivo (*n* = 3). **f** Local magnification in the left kidney of the mouse in part of (**e**). **g** Raw and fitted relative enhancements versus time computed from the quantitative PA signals of ICG-IgG in the kidneys of normal mice and in mice with mild AKI or severe AKI. **h** PA signals in renal cortex of normal mice, mice with mild AKI, and severe AKI at different time points in vivo (*n* = 3). **i** PA signals in renal medulla of normal mice, mice with mild AKI, and severe AKI at different time points in vivo (*n* = 3). **j** PA signals in pelvis of normal mice, mice with mild AKI, and severe AKI at different time points in vivo (*n* = 3). **k**–**m** The fluorescence imaging results of ex vivo kidneys with AKI of different degrees. **n**–**p** The PA imaging results of ex vivo kidneys with AKI of different degrees. **q** The H&E staining of kidneys with AKI of different degrees. **r** Immunohistochemical staining of normal kidneys with AKI of different degrees. The red arrows indicate the deposition of IgG. **s**, **t** Renal function indicators, serum creatinine and urea, in normal kidneys and in kidneys with different degrees of AKI (*n* = 3). Scale bar = 5 mm. *p < 0.05

Equal doses of glomerulonephritis circulating immune complex ICG-IgG were injected via the tail vein into mice with no, mild, and severe AKI, respectively. The results clearly demonstrated that the dynamic deposition process of IgG in mouse kidneys was also captured with high-definition quality by SRCPT. Intravital dynamic monitoring results (see Methods) showed that the deposition of IgG in the kidneys of mice with AKI progressive increased with time from IgG injection until 120 min (*P* < 0.0001) (Fig. 4a, c, e). The results suggested that the original kidney injury would lead to the augmentation of IgG deposition. We further obtained clearance parameters of IgG. The U_α_ was 1.10 ± 0.43 min^−1^, 0.52 ± 0.04 min^−1^ and 0.04 ± 0.02 min^−1^ in kidneys of mice with no, mild, and severe AKI, respectively (*P* < 0.001). The E_β_ was 0.17 ± 0.04 min^−1^ in kidneys of mice without AKI. The AUC_0-120 min_ was 4457 ± 223, 5939 ± 549 and 14599 ± 1460 in kidneys of mice with no, mild, and severe AKI, respectively (*P* < 0.001). The E_β_ was 0.17 ± 0.04 min^−1^ in kidneys of mice without AKI. The above results showed that with the aggravation of AKI, U_α_ of IgG in the kidneys decreased while the deposition of IgG in the kidneys increased, which indicated that the uptake and excretion functions of the kidneys had been seriously damaged. The results of the AUC_0-120_ _min_ also suggested that within 120 min, the kidneys of mice in the severe AKI group had the most IgG deposition. After being ingested by the kidney, IgG was difficult to be excreted from the damaged renal tissue and thus retained in the renal tissue, while IgG in normal tissue could be excreted without deposition. Zoomed-in image of a portion of the mouse’s left kidney clearly showed that IgG was mainly deposited in the renal cortex (Fig. 4b, d, f). Statistical analysis (see Methods) showed significant differences of IgG deposition between the no AKI group and the other two AKI groups (both *P* < 0.05). There were also significant differences of IgG deposition between the mild and severe AKI groups (*P* < 0.0001) (Fig. 4g).

Further, we quantified the PA signals from different structural regions of the mice kidneys, including the renal cortex, renal medulla, and renal pelvis. The quantification results of the renal cortex indicated that IgG deposition was most prominent in this region with increasing renal injury (Fig. 4h). Although IgG deposition in the renal medulla also increased with worsening renal injury, it remained primarily localized in the renal cortex (Fig. 4i). Quantitative results from the renal pelvis region showed that normal mice had minimal IgG excretion in the renal pelvis, while mice with mild and severe AKI exhibited IgG excretion in the renal pelvis (Fig. 4j). In general, biomacromolecule IgG is difficult to pass through the glomerular filtration membrane and enter the urine. Therefore, an increase in IgG content in the renal pelvis can indicate glomerular damage and impaired renal function.

Two hours after injection, the kidneys were excised and imaged at 780 and 850 nm, respectively. Then spectral unmixing analysis was performed. The results clearly demonstrated that IgG exhibited the highest renal deposition in mice suffering from severe AKI followed by those with mild AKI, which was also validated by fluorescence imaging ex vivo (Fig. 4k–p). IgG was mainly deposited in the renal cortical region (cyan pseudo-color represented IgG deposition while purple pseudo-color indicated hemoglobin distribution). Compared to fluorescence imaging results, SRCPT offers higher resolution, and clearly delineates the renal anatomy, including the renal cortex, cone, papilla, and calyx (marked by yellow arrows in Fig. 4n–p). In addition, histological analysis of renal tissue slices from different groups of mice also revealed that compared with the normal group (healthy mice), the model group mice exhibited renal tubular dilation and vacuolar degeneration (Fig. 4f). In the group of severe AKI, the kidney injury was significantly aggravated (Fig. 4q). Immunohistochemical staining detected the most glomerular mesangial IgG deposition in kidneys with severe AKI (Fig. 4r). The renal functional results for different mice groups demonstrated that, compared to the normal group, the model group exhibited significantly elevated levels of serum creatinine and urea (Fig. 4s–t).

### SRCPT and PET of a double-labeled [^68^Ga]DFO-IRDye 800CW

**Fig. 5:**
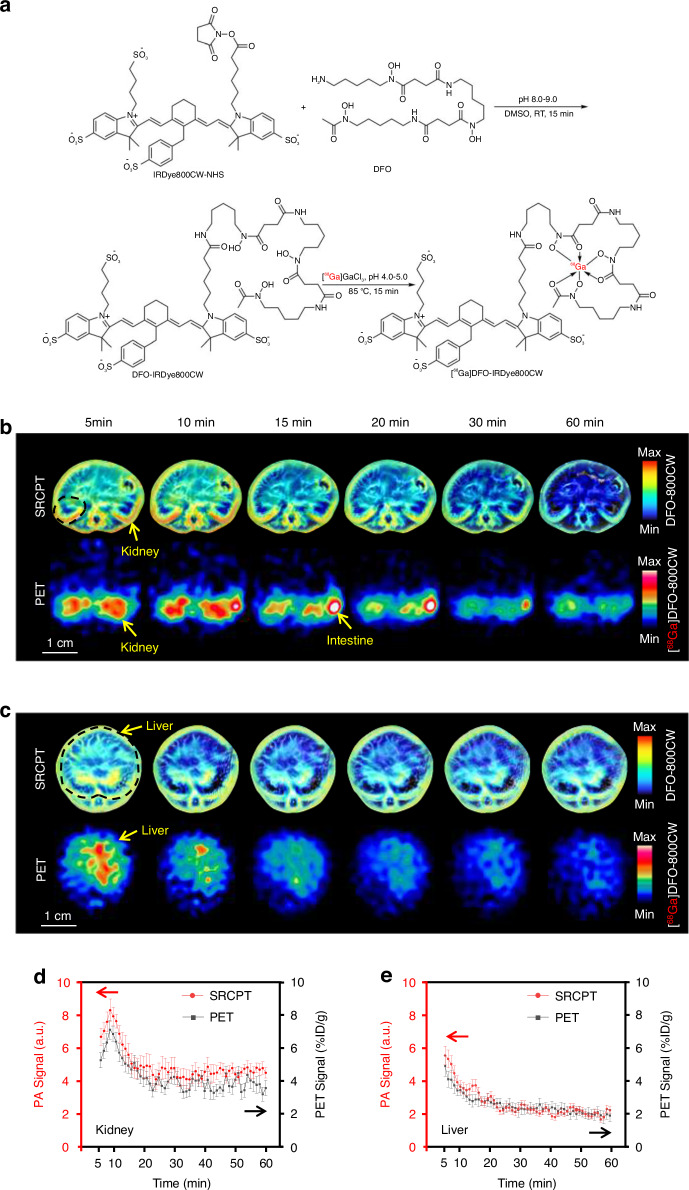
SRCPT and PET of [^68^Ga]DFO-IRDye800CW in mouse kidneys and liver. **a** Synthetic route of a PAT/PET double labeled probe. **b**, **c** Representative metabolic images of SRCPT/PET dual labeled probe at different time points in the kidneys and liver, respectively. **d**, **e** Clearance curves of the double labeled probe at different time points in the kidneys and liver (*n* = 3 in each group), respectively

PET/CT has been widely used for clearance study by employing specific nuclide labeling to the compounds. To validate the precision of SRCPT, we synthesized a [^68^Ga]DFO-IRDye 800CW probe possessing both PAT and PET properties (Fig. 5a). The compound was tested in mice using both SRCPT (774 nm) and PET techniques. Scanning images at different time points showed that the tendency of PAT and PET signals was consistent in the kidneys and liver, but SRCPT depicted much higher spatial resolution (Fig. 5b, c). Subsequently, the quantitative results provided the clearance profile of the probe within the kidneys and liver (Fig. 5d, e). Both SRCPT and PET findings substantiated that the compound primarily underwent renal metabolism, with a minor contribution from hepatic metabolism. Both methods indicated that the peak time (T_max_) within the kidneys was approximately 8 min, while the T_1/2_ was approximately 9 min. Further correlation analysis indicated a strong positive relationship between the two methodologies, as indicated by a Pearson correlation coefficient of 0.967 (*P* < 0.0001). This outcome serves as a validation of the accuracy of SRCPT in relation to PET.

## Discussion

The study introduces an innovative approach, SRCPT, facilitated by the MC-EMM algorithm, aimed at accurately evaluating the concentration mapping and clearance properties of various agents within distinct tissues and organs. The real-time output provided by SRCPT offers immediate visual feedback, allowing researchers to observe the distribution and metabolism of probes promptly. The technique enables informed decisions during imaging sessions, such as optimizing experimental conditions, adjusting drug dosage, or altering imaging parameters to capture specific dynamic events.

The PA imaging contrast primarily depends on optical absorption and absorption-to-heat conversion efficiency. Thus, utilizing endogenous chromophores or selecting suitable contrast agents is crucial for this technology^27,28^. Common chromophores such as melanin, oxyhemoglobin/deoxyhemoglobin, lipids, and collagen can directly provide valuable information for clinical diagnostics. Similarly, exogenous agents with high optical absorption and quantum yield possess a natural imaging advantage in SRCPT.

To ensure the effectiveness of SRCPT, particularly in cases where chemical substances lack sufficient optical absorption, appropriate labeling of optical tracers is required. The labeled product should closely resemble the original chemical in polarity and size to maintain previous pharmacokinetic properties, necessitating further validation through rigorous biochemical experiments. If the biochemical properties of the labeled compounds cannot be assured, we do not recommend using SRCPT for monitoring.

Additionally, we prefer clinically approved drugs with good photothermal effects and contrast agents specifically designed for PA imaging. We have already validated the effectiveness of mitoxantrone as the former. Representative contrast agents in the latter category include gold nanocrystals, carbon nanotubes, graphene-based agents, 2D graphene analogs, organic nanoparticles, and semiconductor polymer nanoparticles. These agents not only possess excellent photostability and biophysical properties but also offer diagnostic, monitoring, and therapeutic functionalities. Our developed SRCPT technology can further explore combined applications with different drugs.

In this study, taking into account the factor of light attenuation, we introduced the variable of light flux into the MC-EMM algorithm. This algorithm considers the different absorption and scattering of different tissues, making it more adaptive for SRCPT. The MC-EMM algorithm, adapted from empirical mathematical models used in MRI data analysis, is tailored to calculate pharmacokinetic changes in drugs sampled at a rate as fast as 0.05 s, considering factors like light attenuation in tissues. The algorithm enhances the capability of SRCPT to map the clearance pathway of agents in organs accurately, facilitating the assessment of parameters in multiple critical sites simultaneously. However, there are also limitations: it requires prior knowledge of optical parameters for ROIs, such as scattering coefficients. Thus, estimation errors in optical parameters across different ROIs can affect the fitting accuracy of MC-EMM. Despite these parameter effects, optical parameters with errors can still yield more accurate fitting results. This precision arises from the ratio of photon flux distributions at times t_0_ and t, indicating relative changes in magnitude.

Through analysis of these parameters, we can distinguish the main organs involved in the metabolism of these probes. In this way, the distribution and metabolism of agents can be observed once the agents enter the body, and quantitatively analyze the metabolic processes in various tissues, so as to judge the action site of agents and possible toxic side effects.

Our findings also provide valuable insights into the clearance pathways of the clinical drug mitoxantrone, particularly in the context of normal physiological conditions and acute organ injuries. Through SRCPT at a 3D level, the study reveals the dynamic clearance process of mitoxantrone in mice, offering significant advancements in drug metabolism research. Additionally, the study employs a novel FCS-SAFT algorithm for 3D PA imaging, enhancing resolution in both lateral and elevational directions, providing a clearer visualization of drug clearance pathways. Our findings emphasize the critical role of SRCPT combined with advanced algorithms in precisely tracking drug clearance pathways across different organs and pathological conditions. These insights offer valuable implications for drug metabolism research and potential therapeutic interventions. The use of advanced imaging techniques, such as 3D imaging and innovative algorithms, contributes to the accuracy and resolution of the results, further establishing SRCPT as a promising tool for future drug tracking studies.

In conclusion, intravital SRCPT with the MC-EMM algorithm proves to be an ideal tool for thorough understanding of the clearance pathways of probes, especially within crucial metabolic organs like the liver and kidneys. This innovative technique not only provides real-time insights into drug clearance but also paves the way for advancements in precision medicine. Its introduction opens up new avenues for further research and development in the field, promising significant contributions to biomedical science and healthcare^29^.

## Materials and methods

### PAT configuration

The hardware part schematic for SRCPT is depicted in Fig. 1a. We firstly expanded the laser beam from a wide-band laser (Q-smart 450, Quantel laser, France) by a concave lens (LD1357, Thorlabs, USA) then made it in a conical-surface pattern by a conical lens (AX-FS-1-140-0, Del Mar Photonics, USA). A water tank was used to submerge the target in the water for acoustic coupling. The ring-shape laser beam was then refocused by a home-made optical condenser to form a ring pattern on the trunk of mice, with the illuminated area positioned in the elevational focal zone of transducer array. The laser repetition rate was 20 Hz with wavelengths ranging from 680 to 950 nm, 1064 nm, and 1190 to 2600 nm output. A full-ring ultrasonic transducer array with 512 elements (Imasonic, France) was employed for high-speed PA signal detection. The diameter of the ultrasonic ring array was 100 mm with a central frequency of 5.5 MHz. A 512-channel data acquisition system (Photosound, USA) with 40 MSa s^−1^ sampling rate was subsequently used to receive PA signals simultaneously in 512 parallel channels. Compared with the PA computed tomography system previously reported by Li et al.^30^, the image reconstruction is accelerated by parallel computing in C language to simultaneously display images in the meantime of capturing original data in this study. Specifically, we employed CUDA to harness the power of graphics processing units (GPUs) for parallel computing the delay of each channel. Moreover, we have upgraded the laser with much higher energy and optimized the device of mice mount, making it much easier for orientation adjustments of mouse. We used a breathable tape to secure the upper part of the mouse, ensuring minimal movement during imaging. This approach helped to eliminate motion artifacts and enhance the stability of the imaging process. Additionally, we incorporated a mouse lifting device that allowed us to adjust the step distance according to the specific experimental requirements, further optimizing the imaging setup for small animal studies.

### Animal preparation and surgery

In this work, 8-week-old male nude mice (SPF biotechnology Co., Ltd. Beijing, China) were used for in vivo experiments. The mouse was anesthetized by vaporized isoflurane (1 MAC), and the body of mice, except the mouth and nose, was immersed in water at 36 °C. All experimental procedures followed laboratory animal protocols approved by the institutional animal care and use committees in Guangdong Provincial People’s Hospital.

For the APAP-ALI model, we fasted the mice for 16–18 h prior to APAP administration to potentiate hepatotoxicity. We dissolved APAP in sterile saline solution to create a concentrated stock solution and administered APAP (300 mg kg^−1^) by intraperitoneal injection. SRCPT was performed on the mouse 4 h after APAP injection.

To establish a model of AKI at different stages, mice were intraperitoneally injected with cisplatin at three different dosages. The normal mice group was injected with 300 μL of normal saline. The mild AKI group was injected with 300 μL of cisplatin at 20 mg kg^−1^ while the severe AKI group was injected with 300 μL of cisplatin at 20 mg kg^−1^ for 3 days continuously.

### Agent preparation

Herein, three different dyes, A1094, ICG (S46424, Shanghaiyuanye Bio-Technology Co., Ltd, China) and IRdye800CW (929–70020, LI-COR Biosciences, USA), were used as optical contrast agents. Ultrapure water was used as the solvent for a maximum concentration of 100 nM for all dyes. All dyes injection does was 1 μmol per kilogram of body weight. As a clinical drug, mitoxantrone (HY-13502, MedChemExpress, USA) was evaluated for its clearance parameters in the liver and kidneys. It was dissolved in sterile saline solution and administered to mice via tail vein at a dose of 2 mg kg^−1^.

To obtain ICG-IgG, human mono-IgG purified protein (ab205806, Abcam, USA) and ICG-NHS Ester (R-TE-157; Ruixibio, China) were stirred at room temperature in the dark for 4 h (pH 8-9), and the excess solvent DMSO was removed by dialysis against PBS buffer. The molar ratio of ICG and protein in the labeling process was 10:1. The unconjugated ICG was removed by gel filtration using a Zeba spin desalting column (89890; Thermo Fisher Scientific, USA). Microplate readers (Spark, Tecan Austria GmbH, Austria) were used to obtain the absorption curve of the target sample at 300–1000 nm. 20 nmol ICG-labeled IgG protein was injected into the tail vein of mice.

The final solutions were injected through the caudal vein with a 1 mL syringe connected to the catheter by a 25-G needle. The solutions were injected by a syringe pump (D107886, KD Scientific Inc., USA).

To obtain [^68^Ga]DFO-IRDye800CW, DFO-IRDye800CW was synthesized through the reaction of NHS ester and amine as the precursor of the double labeled probe. IRDye800CW Ester and DFO (HY-B0988, MedChemExpress, USA) were stirred at room temperature in the dark for 15 min (pH 8–9). The excess solvent DMSO was removed by dialysis against PBS buffer. [^68^Ga]DFO-IRDye800CW was ^68^Ga-labeled through mixing [^68^Ga]Ga^3+^ with DFO-IRDye800CW and adjusting the pH to 4–5 at 85 °C in the dark for 15 min. The probe was then examined for SRCPT and PET on mice.

### Fluorescence imaging

The labeled IgG distribution was captured by in vivo fluorescence imaging (Perkin Elmer, USA). In different renal injury models, the kidneys were removed after euthanasia two hour after IgG injection. Fluorescence images were captured under 789 nm laser excitation.

### PET/CT imaging

MicroPET/CT of [^68^Ga]DFO-IRDye800CW was performed on an Inveon PET/CT scanner (Siemens Medical Solutions Inc., USA). Images were reconstructed iteratively using 3D OPMAP 128.pPetRcn (Siemens Medical Solutions Inc., USA) and converted to %ID g^−1^ images. Doses of intravenously injected [^68^Ga]DFO-IRDye800CW were calculated utilizing each mouse’s weight (10 μCi g^−1^). After [^68^Ga]DFO-IRDye800CW injection, the mice were continuously scanned for one hour.

### Laser wavelength selection

Specific wavelengths covering NIR I&II can be selected for different application scenarios. Here, we used 1064 nm for structural imaging since hemoglobin has a lower absorption at this wavelength, allowing imaging into deeper tissues. Additionally, 1064 nm laser was used to track the A1094 dye owing to its peak absorption wavelength. For liver/kidney functional oxygenation imaging, wavelengths of 750 and 850 nm were used since the molar optical absorption of deoxyhemoglobin and deoxyhemoglobin have a large difference at these two wavelengths. The absorption peaks of ICG/ICG-IgG and IRDye 800CW were 780 and 774 nm, respectively.

### Image processing for dynamic tracking

To reconstruct the PA images from the 512-channel raw signals, the half-time dual-speed-of-sound universal back-projection algorithm^44^ was employed with a pixel size of 25 μm. The contrast of the reconstructed image was then enhanced by applying Hessian-based Frangi vesselness filters^31^. Of note, the quantitative analyses were calculated by raw reconstructed image without image enhancement.

The first 600 frames of the PA image were averaged as the baseline image before injecting the probe. Next, the organs were continuously monitored during probe circulation. To track the distribution of probes, a 2D adaptive noise-removal filter^32^ was used to denoise the PA images; subsequently, each frame was subtracted from the baseline image to obtain the differential image. The interference was further suppressed by thresholding the pixel values at 20% of maximum. Finally, the differential image was merged to the enhanced PA image as pseudo-color.

### FCS-SAFT

FCS-SAFT utilizes the synthetic aperture focusing technique in ultrasound imaging to address the defocusing issues of ultrasound signals that are far from the acoustic focus, thereby enhancing the resolution of 3D data. This algorithm integrates the scanning parameters from SIP-PACT for calculations.

Using data acquired from a single ultrasound transducer as an example, the acoustic signals excited by the target material far from the focal zone tend to spread to other sampling layers during propagation. This propagation range z can be expressed by a linear function:

$$z=\pm \left(l-{l}_{f}\right)* \frac{w}{2{l}_{f}}$$

In this formula, $l$ is the distance from the target to the transducer, ${l}_{f}$ is the distance from the focus to the transducer, and $w$ is the height of the transducer.

In practical calculations, the coherent range is constrained to a certain number of coherent layers ${z}_{s}$ using the scan step size $\Delta z$:

$${z}_{s}=\left\lfloor \frac{z}{\Delta z}\right\rfloor$$

The position of the defocused artifacts at the target detection point in the coherent layers can be determined by calculating the propagation time of the acoustic signal. The time delay difference $\Delta {t}_{j}$ in the coherent layers j can be expressed as:

$$\Delta {t}_{j}={sign}\left(l-{l}_{f}\right)* \frac{{r}_{i+j}-{r}_{i}}{{c}_{{water}}}$$

Here, ${c}_{{water}}$ is the speed of sound in the medium. ${r}_{i}$ represents the distance between the target detection point and the focal point, while ${r}_{i+j}$ represents the distance between the projected position of the target detection point in the $j$ layer and the focal point. This relationship can be expressed as:

$${ri}=\left|\frac{{n}_{0}-{n}_{i}}{{f}_{s}}* {c}_{{water}}\right|$$

$${r}_{i+j}=\sqrt{{r}_{i}^{2}+{\left(j* \Delta z\right)}^{2}}$$

(${n}_{i}$ is the target sampling point, ${n}_{0}$ is the focal point, and ${f}_{s}$ is the sampling frequency).

The synthetic aperture process for the target point can be understood as focusing the defocused parts. Therefore, the PA data processed by SAFT, ${{PA}}_{{SAFT}}$ can be expressed as:

$${{PA}}_{{SAFT}}=\mathop{\sum }\limits_{j=-\frac{{z}_{s}}{2}}^{\frac{{z}_{s}}{2}}{PA}\left(i+j,t-\Delta {t}_{j}\right)$$

During the synthesis process, noise present in the data across different layers can significantly affect the final synthesis result. Therefore, a coherence factor (CF) is needed to constrain the PA data. For a set of PA data to be synthesized, the ${CF}$ value can be determined based on its degree of dispersion:

$${CF}=\frac{{{{PA}}_{{SAFT}}}^{2}}{{z}_{s}* \mathop{\sum }\nolimits_{j=-\frac{{z}_{s}}{2}}^{\frac{{z}_{s}}{2}}{\left|{PA}\left(i+j,t-\Delta {t}_{j}\right)\right|}^{2}}$$

The final weighted SAFT-corrected data is obtained after applying the weighting factor correction:

$${{PA}}_{{CFSAFT}}={{PA}}_{{SAFT}}* {CF}$$

The corrected PA data, after spectrum separation, can achieve the separation of high-frequency vascular signals and low-frequency tissue signals.

### Histopathological staining

Immunohistochemical staining: H&E staining and Masson staining were performed according to standard procedures. Tissue sections were fixed with 4% paraformaldehyde for 5 min, washed with PBS, blocked with blocking solution (X0909; Dako) for 1 h, and incubated with primary antibody overnight at 4 °C. After washing with PBS, the slides were incubated with secondary antibody for 1 h at room temperature. Antibodies used for immunohistochemical staining were as follows: rabbit anti-human IgG (ab109489, Abcam, USA) and Goat Rabbit IgG (HRP conjugate) (511203, ZEN BIO, China).

### Statistical analysis

SPSS Version 24 Software (IBM Corp, USA) was used for statistical analysis. Quantitative data were expressed as mean ± SD. Pearson correlation analysis was used to analyze the correlation between SRCPT and PET. For the analysis of IgG deposition in the kidneys of mice in the different model groups, repeated ANOVA measurement was used to compare the differences between groups. Following Mauchly’s Test of sphericity of the data, intra-group effect analysis was conducted by using the correction results of Greenhouse-Geisser. When the data met the hypothesis of homogeneity of variance, the LSD test was used for multiple comparison analysis between groups. When the data did not meet the hypothesis of homogeneity of variance, the Dunnett’s T3 test was used for multiple comparison analysis between groups. A two-tailed *P* value < 0.05 was considered statistically significant.

## Supplementary information

Supplementary Video 1

Supplementary Video 2

Supplementary Video 3

Supplementary Video 4

Supplementary Video 5

Supplementary Video 6

Supplementary Video 7

## Data Availability

The data supporting this study's findings are available from the corresponding author upon reasonable request.
